# Restoring Eelgrass (*Zostera marina* L.) Habitats Using a Simple and Effective Transplanting Technique

**DOI:** 10.1371/journal.pone.0092982

**Published:** 2014-04-02

**Authors:** Yi Zhou, Peng Liu, Bingjian Liu, Xujia Liu, Xiaomei Zhang, Feng Wang, Hongsheng Yang

**Affiliations:** 1 Key Laboratory of Marine Ecology and Environmental Sciences, Institute of Oceanology, Chinese Academy of Sciences, Qingdao, P. R. China; 2 University of Chinese Academy of Sciences, Beijing, P. R. China; 3 Guangxi Institute of Oceanology, Key Laboratory of Marine Biological technology, Beihai, P. R. China; The University of Adelaide, Australia

## Abstract

Eelgrass beds in coastal waters of China have declined substantially over the past 30 years. In this study, a simple new transplanting technique was developed for eelgrass (*Zostera marina* L.) restoration. To assist in anchoring single shoots, several rhizomes of rooted shoots were bound to a small elongate stone (50–150 g) with biodegradable thread (cotton or hemp), and then the bound packet was buried at an angle in the sediments at a depth of 2–4 cm. This stone anchoring method was used to transplant eelgrass in early November 2009 and late May 2010 in Huiquan Bay, Qingdao. The method led to high success. Three month survivorship of the transplanted shoots at the two transplant sites was >95%. From April 20 to November 19, 2012, the following characteristics of the 2009 and 2010 transplanted eelgrass beds were monitored: morphological changes, shoot density, shoot height, leaf biomass, and sediment particle size. Results showed that the sexual reproduction period of the planted eelgrass was from April to August, and vegetative reproduction reached its peak in autumn. Maximum shoot height and biomass were observed in June and July. After becoming established, the transplanted eelgrass beds were statistically equal to natural eelgrass beds nearby in terms of shoot height, biomass, and seasonal variations. This indicates that the transplant technique is effective for eelgrass restoration in coastal waters.

## Introduction

Seagrasses, a unique group of marine submerged angiosperms, are widely distributed along temperate and tropical coastlines of the world, and they have high productivity and biodiversity and provide great ecological and economic values [1]–[3]. Seagrasses are prominent marine ecosystem engineers, or foundation species in many coastal waters, as they can significantly modify abiotic environment [4]–[5]. They can attenuate hydrodynamic energy from currents and waves, stabilize the seabed sediments, accelerate the sedimentation of the suspended particles and purify seawater, and provide habitats, nursing grounds and food for marine animals [6]–[8]. Recently, seagrass beds have been suggested to be key sites for global carbon storage in the biosphere [9]–[11]. However, seagrass beds are globally disappearing at an alarming rate due to anthropogenic influences [12]–[14].

Increased interest in seagrass restoration in recent decades has resulted in the development of various transplantation methods using either seagrass shoots (adult plants or seedlings) or seeds [15]–[27]. Adult plants with bared rhizomes and roots, either anchored [16], [22] or unanchored [17], have been commonly used in eelgrass transplantation efforts. Seed sowing is considered to be an economically effective method for large-scale restoration [28]. However, the seed-broadcast technique is only useful when seeds can settle and germinate, such as in areas with low seed predation and little physical disturbance [22]. Also, manual seed collection and sowing are labor intensive because mechanical collection and sowing of seeds are not suitable in some places. Transplantation of adult plants is the simplest and most common method, and its use of the vegetative reproduction process makes it very effective. In addition, low seedling establishment rates have been reported in some areas [29]. To date, several techniques for transplanting adult plants have been shown to be successful in establishing seagrass populations [16]–[17], [19], [22], [30]–[32], and they can be divided into two categories. The first involves transplanting shoots with sediment intact (i.e., plugs/cores or sods), which can minimize disruption of roots and rhizomes [19], [21] but may cause great damage to the donor bed and requires enormous manpower and time [30]. The second category involves transplanting seagrass shoots with bare roots; this approach is more environmentally friendly, but it generally requires anchoring of the shoots [16]. The stone binding method developed in the present study belongs to the latter category. To determine whether a transplanting effort is successful or not, long-term monitoring (e.g. several years) is necessary; while traditional short-term monitoring (i.e., <1 year) may lead to a biased result [27]. From the first European Seagrass Restoration Workshop, Cunha et al. [27] point out that few seagrass restoration programs developed in Europe by the participants during the last decade are successful; and most projects reporting success are biased by the fact that most of them had very short-term monitoring (i.e.<1 year). Restoration success or failure may be linked to differences between years in physical or biological factors, such as climate/weather issues, herbivory, macroalgae, or exposure. A stressor may be absent in a year but present the next.

The eelgrass *Zostera marina* L is the dominating seagrass species in the Northern Hemisphere. It is also the most widespread seagrass in northern China. It grows into large-scale communities in shallow coastal waters including intertidal zone [33]. In China eelgrass has decreased greatly since the 1970s, owing to human-induced habitat deterioration. Increasing human activities are generally considered to be responsible for this reduction. However, on a more positive note, the public understanding of the importance of eelgrass in ecosystem functioning has been enhanced in recent years.

In this study we developed a simple new transplantation method for *Z. marina* restoration. We transplanted *Z. marina* using the stone anchoring method, and we then monitored the growth characteristics of the transplanted eelgrass 2–3 years after transplantation. Characteristics of the restored bed were compared with those of natural seagrass bed to verify the effectiveness of this method. Our main hypothesis was that, after 2–3 years of adult-plant transplantation using the stone anchoring method, eelgrass meadow at the transplant sites was equal to the nearby natural meadow.

## Materials and Methods

### Ethics Statement

No specific permit was required for collecting of the eelgrass *Zostera marina* L. from Huiquan Bay and Qingdao Bay, and for transplanting of the eelgrass in Huiquan Bay, Qingdao. The field studies did not involve endangered or protected species.

### 2.1. Study Sites

**Figure 1:**
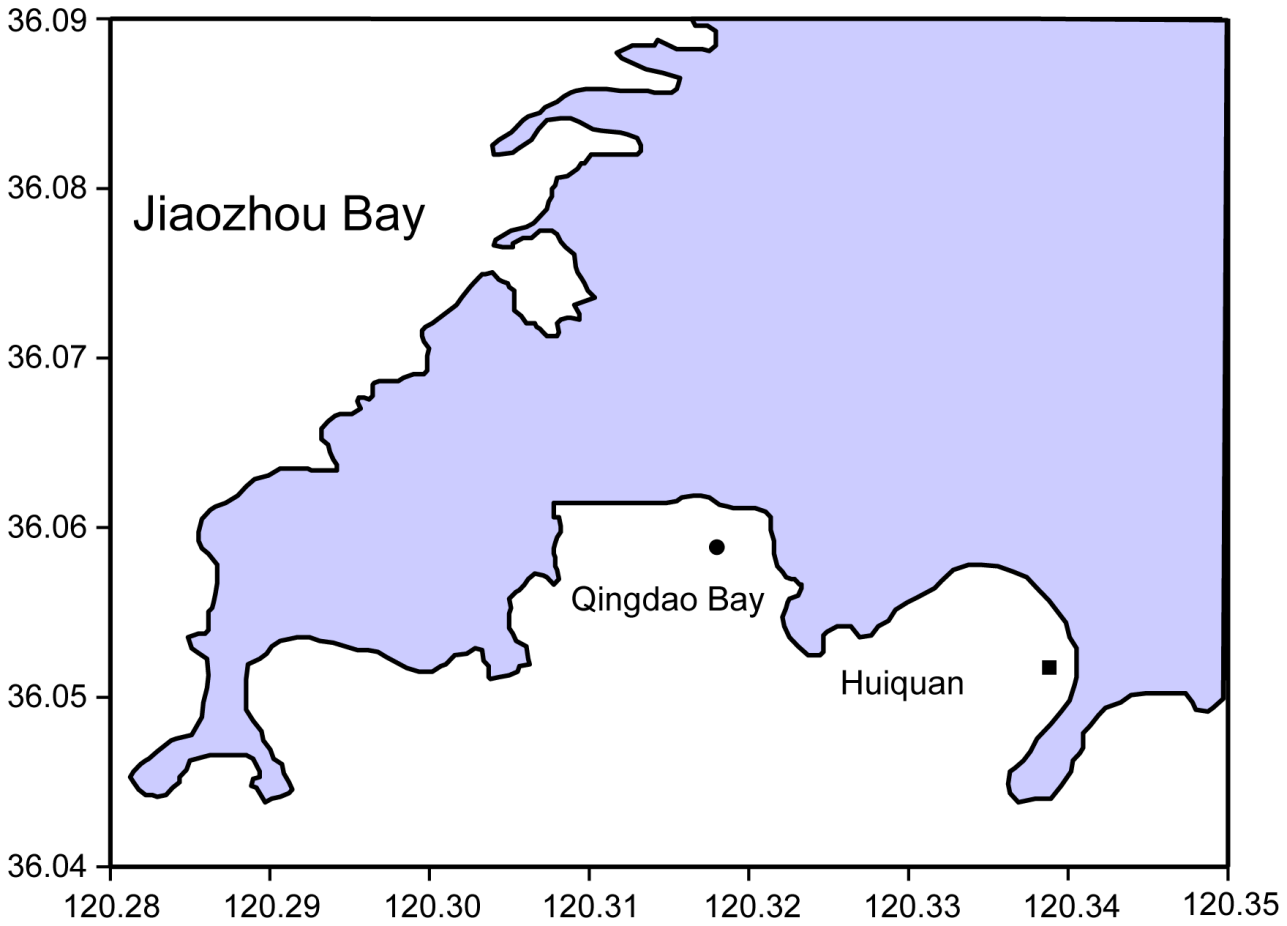
Location of study sites. ▪, eelgrass transplantation zone in Huiquan Bay; •, natural intertidal eelgrass bed in Qingdao Bay.

The experiment was conducted in Huiquan Bay (120.339°E, 36.053°N; transplant site) and Qingdao Bay (120.318°E, 36.059°N; reference site) in Qingdao City, Shandong Province, China (Figure 1). Qingdao is located in the north temperate monsoon zone. The bays are located in the south of Qingdao City, and both are open gulfs with a semidiurnal tide. A natural eelgrass bed is present in Qingdao Bay. There once was an extensive seagrass bed extending from the intertidal to the subtidal zone in Huiquan Bay, but currently only a small area of natural eelgrass remains, and it occurs mainly in the subtidal zone. Although the specific reason for dramatic decline of the seagrass in Huiquan Bay is unknown, the human-induced habitat deterioration and water pollution in the last several decades of the 20th century might be responsible for this reduction. However, coastal water environment in Qingdao has been improved in recent years due to local efforts on coastal conservation and management.

### 2.2. Collection of adult plants

In early November (late autumn) 2009, adult plants were collected from the intertidal area in Huiquan Bay at low tide. A storm and strong waves transported these plants to the intertidal from the subtidal zone of the bay the day before they were collected. Fresh rooted shoots that had at least 1–2 cm of rhizome with roots were selected. Twenty centimeters of leaf blade and leaf sheath were retained, and the extra part was removed by scissors. Decaying leaves were also removed.

In late May (late spring) 2010, adult plants with rhizomes and roots were collected from the intertidal wild population by hand at Qingdao Bay (Figure 1). Hand collection of 1–3 eelgrass shoots at a time minimized disruption of the donor site. Collectors followed shoot blades to the substrate, uprooted ca. 3–5 cm of rhizomes by digging under them by hand, and snapped the rhizomes to remove the plants. Rooted adult plants were sorted out based on rhizomes and leaf sheaths, using the same method as described above.

### 2.3. Transplanting method

Eelgrass shoots were planted in early November (late autumn) 2009 and in late May (late spring) 2010 in intertidal zone of Huiquan Bay after the plants were collected and prepared. Specimens were transplanted 25 cm apart within a row, and distance between rows was 25 cm. Eelgrass shoots were transplanted using a stone anchoring method. This method involves anchoring a transplanting unit (PU) consisting of three shoots with rhizomes and roots to a small elongate stone of 50–150 g using biodegradable thread or thin rope (e.g., cotton thread or hemp string). The small stones were collected from the sea shore in Huiquan Bay. At the transplant sites, PUs were buried in holes dug with a scoop so that the rhizomes were situated at a depth of 2–4 cm in sediments and on the side of the stone. The final position of the plants was similar to that which occurs naturally. The buried rhizomes were parallel to the sediment surface, but the shoots were inclined at an angle toward the shore or towards the prevailing wave direction to minimize disruptions caused by strong waves. In the wide intertidal zone, the transplantation is suitable at low tide every month. The total intertidal transplant areas in 2009 and 2010 were 800 m^2^ and 900 m^2^, respectively. Compared with the 2009 transplant site, the 2010 site was at a somewhat greater water depth. The interval between the two transplant sites was 1 m.

In addition, we tested a modified stone anchoring method for eelgrass transplantation in the subtidal zone. This simplified transplant method involved simply placing each stone-anchored PU on the surface of the tidal flat. In this case, the PU rhizomes were mostly situated underneath the stone and thus were touching the sediment. On November 4–5, 2009, eight 1×1 m^2^ plots were planted using the simplified method. In each plot, specimens were transplanted 25 cm apart with a row interval of 25 cm. The distance between adjacent plots was 1 m.

### 2.4. Eelgrass monitoring

Transplant survivorship at the 2009 and 2010 transplant sites in Huiquan Bay was calculated monthly during the first 3 months after transplantation, with all transplanted PUs counted. After this time point, the transplanted plants were observed by eye every 3 months. We found that the transplanted eelgrass was all in good condition. Transplant survivorship in the plots created using the simplified transplant method was also calculated.

Monthly monitoring of the transplanted eelgrass at the 2009 and 2010 sites was conducted from April 20 to November 19, 2012, and the water temperature also was measured. Shoot density (shoots m^−2^) at the transplant sites was measured using a 30 cm ×30 cm quadrat, with 5–6 replicates randomly selected. Shoot height (cm) and the aboveground biomass (dry weight (DW); g DW m^−2^) were measured by collecting the entire aboveground part of the plant (4–5 replicates). C and N contents of eelgrass leaf collected on June 19, 2012 at the 2009 site were measured using a VARIO EL III elemental analyzer, and P content was measured using the Solórzano and Sharp [34] method modified for particulate total P determination [35]. At the 2009 and 2010 sites, together three replicate sediment samples (collected to a depth of 15 cm using a shovel) were collected, and the grain size distribution of the sediments was determined. For the reference meadow, the shoot density, shoot height and the aboveground biomass of the natural intertidal eelgrass bed in Qingdao Bay were measured on 21 June 2009 (before adult-plant collection) and 19 June 2012 (after adult-plant collection) using the same method as described above.

Results are presented as mean ± SD. Temporal differences in biological and environmental variables were tested using one way analysis of variance (ANOVA). Differences between sites (the two transplanted plots using the standard stone-anchoring method, the transplant site using the modified stone-anchoring method, and the natural eelgrass bed in Qingdao Bay) in biological variables at the same sampling time were also tested using one-way ANOVA. Prior to analysis, data were examined for homogeneity of variances using Levene's tests. Differences were considered significant at a probability level of p≤0.05. Statistics were performed using SPSS 16.0 software.

## Results

For first 3 months after transplantation, plant survivorship at the 2009 site averaged 96.5±3.4%, 98.3±1.6%, and 97.6±2.5%, respectively; and at the 2010 site, averaged 97.8±1.4%, 98.2±1.8%, and 99.0±1.0%, respectively. Through field observations, the main seasonal variations in morphology of the transplanted eelgrass during the experimental period were recorded (Table 1). In mid-May 2011, the transplanted eelgrass plants in both the 2009 and 2010 areas were almost coalescing within and between rows, and it became impossible to identify and count the original shoots individually. In April 2012, reproductive shoots appeared, and the height was obviously higher than that of vegetative shoots. Leaf sheaths of reproductive shoots became thinner and longer and started to form inflorescences without blossoms. In May, the reproductive shoots bloomed extensively. The percentage of reproductive shoots in the transplanted eelgrass beds was the largest at this time point (27.5%). In June, seeds began to form in the reproductive shoots, but they were not yet mature. At the beginning of July, most seeds had matured and shed naturally. In the middle of July, the reproductive shoots began to decay and the density of reproductive shoots began to decline. In August, the reproductive shoots totally disappeared, and epiphytic algae attached to the eelgrass leaves increased. In the middle of September, the number of lateral shoots increased significantly, indicating that the eelgrass had started vegetative reproduction. In late September, the new lateral shoots grew. In October, macroalgae began to attach to the leaves of the new shoots. In November, old shoots defoliated and some disappeared. They were replaced by new shoots with less algae attached to them.

**Table 1:** Main morphological features of the transplanted *Zostera marina* monitored in 2012.

Date	Water temperature (°C)	Morphological feature
April 19	9.4	Reproductive shoots appearing
May 19	13.3	Reproductive shoots blossoming
June 5	17.7	Seeds forming
June 19	18.9	Seeds had formed
July 9	22.5	Most seeds mature
July 19	23.4	Reproductive shoots disappearing
Aug. 23	24.7	Reproductive shoots had disappeared; amount of attached macroalgae increasing
Sept. 16	26.1	Number of lateral shoots increasing; old leaves covered with macroalgae
Sept. 27	23.3	New shoots growing out, old leaves defoliating
Nov. 1	14.6	Old leaves had disappeared, shoot height becoming lower
Nov. 19	7.8	Most attached macroalgae disappeared

Based on atmospheric-temperature data from Qingdao in 2012, the seasons during 2012 were as follows: winter from January 1 to April 3 (<10°C), spring from April 4 to June 21 (10–22°C), summer from June 22 to September 26 (>22°C), autumn from September 27 to November 9 (10–22°C), and winter from November 10 to December 31 (<10°C). The variations in the characteristics of the transplanted eelgrass followed a certain seasonal pattern. In spring when the water temperature was optimal, the eelgrass began sexual reproduction. The seeds matured in summer when the water temperature was higher. In autumn, vegetative reproduction began due to the lower water temperature. The lateral shoots grew fast, the leaves of old shoots defoliated, and the amount of algae attached to the leaf blades increased. In winter, the amount of attached algae declined.

**Figure 2:**
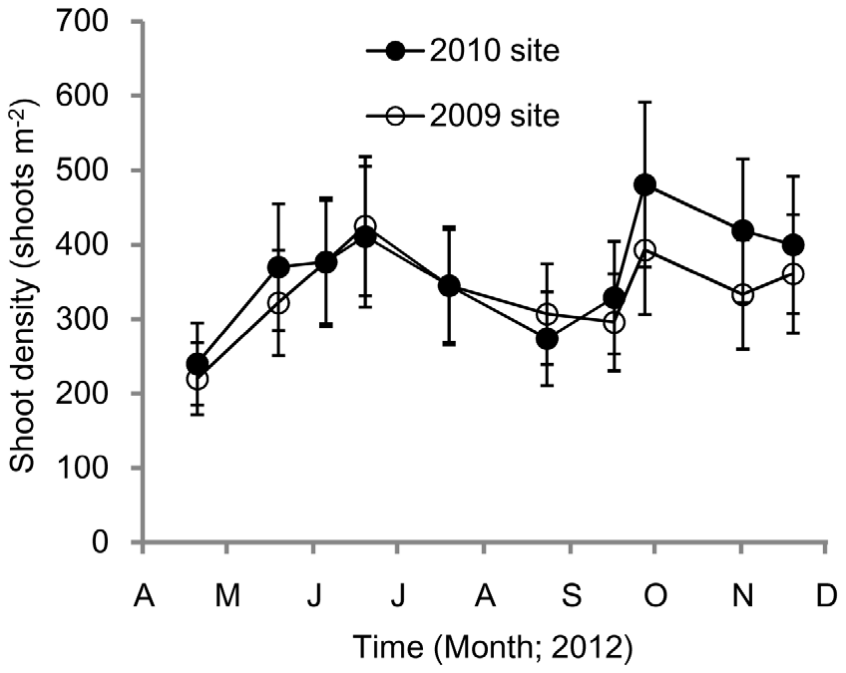
Variations in shoot density (shoots m^−2^) of *Zostera marina* at the 2009 and 2010 transplant sites. Values are means ± SD.

During the 2012 evaluation period, shoot density of transplanted eelgrass ranged from 220 to 481 shoots m^−2^ (Figure 2). Density of the plants at the 2009 transplant site peaked in the middle of June (425 shoots m^−2^) and in late September (393 shoots m^−2^). For the 2010 transplant site, the density peaks also appeared in the middle of June (411 shoots m^−2^) and late September (481 shoots m^−2^). The water temperature was 18.9 °C in mid-June and 23.3°C in late September. Shoot density of the eelgrass increased in spring and peaked in summer, when the water temperature was above 20 °C and the seeds matured and shed; and then with the disappearance of the reproductive shoots, total shoot density was thus decreased. In autumn, the water temperature declined, and the lateral shoots appeared quickly, leading to an increase in total shoot density.

**Figure 3:**
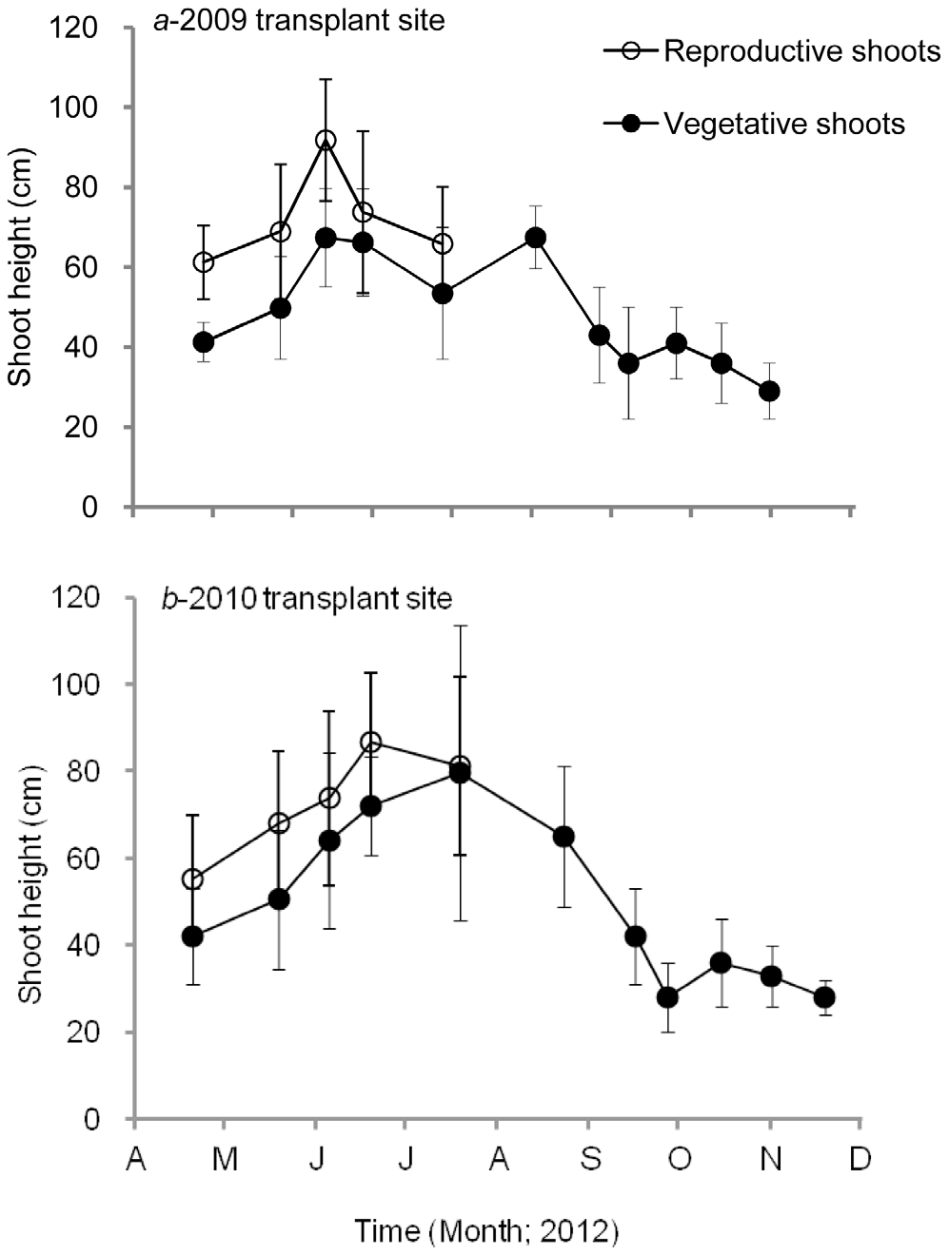
Variations in shoot height (cm) of *Zostera marina* at the 2009 (*a*) and 2010 (*b*) transplant sites. ○, reproductive shoots; •, vegetative shoots. Values are means ± SD.

**Figure 4:**
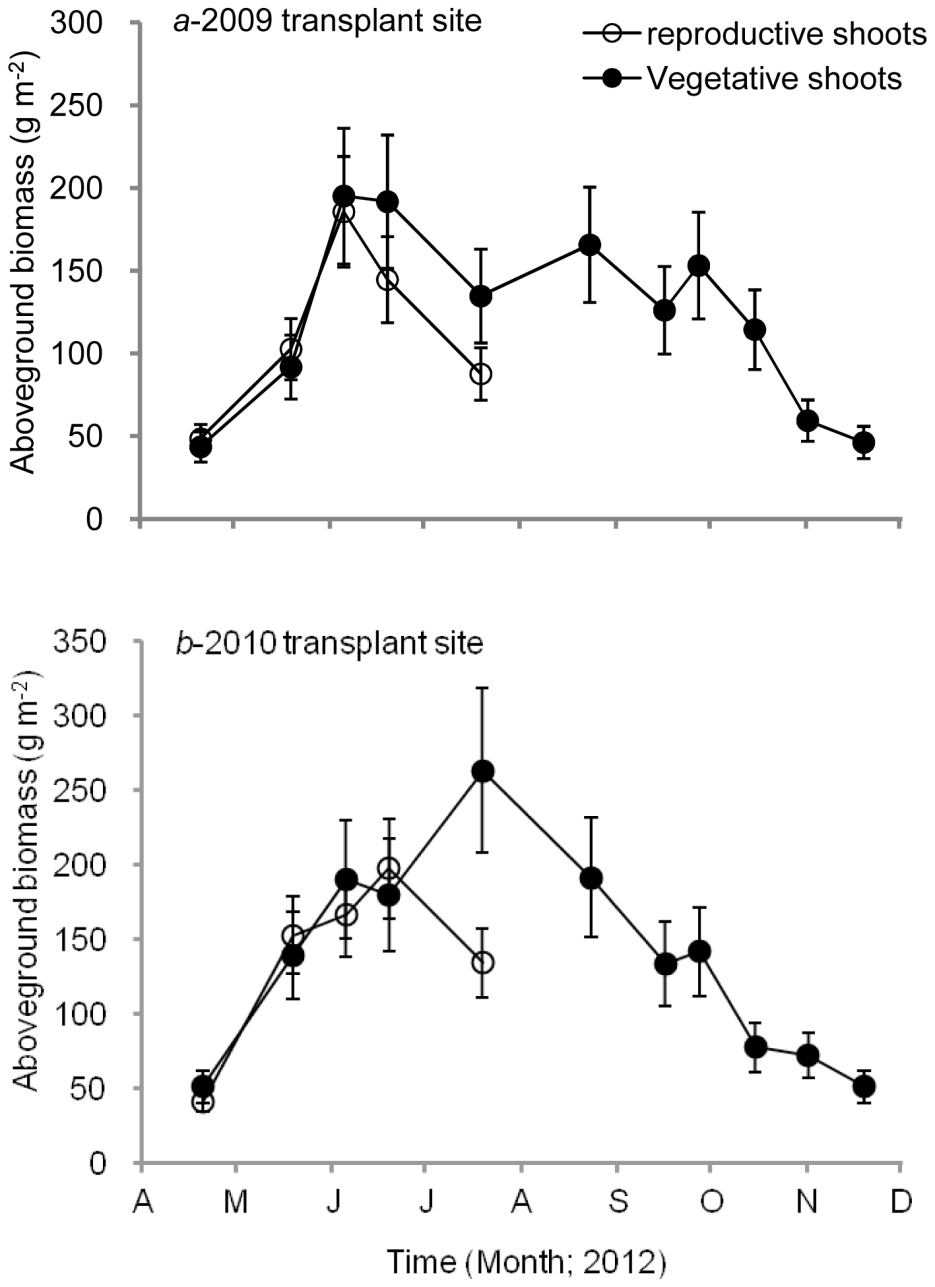
Variations in aboveground biomass of *Zostera marina* (g DWm^−2^) at the 2009 (*a*) and 2010 (*b*) transplant sites. ○, reproductive shoots; •, vegetative shoots. Values are means ± SD.

For the 2009 transplant site, the minimum average height of reproductive shoots (61.3±9.2 cm) was found in April, whereas the maximum occurred in June (91.8±15.2 cm) (Figure 3). The maximum average height of vegetative shoots occurred in August (67.5±7.9 cm). For the 2010 transplant site, the minimum average height of reproductive shoots was found in April (55.2±14.6 cm) and the maximum value occurred in June (86.7±15.9 cm). The maximum average height of vegetative shoots occurred in July (79.6±33.9 cm). The maximum aboveground biomass of reproductive and vegetative shoots at the 2009 transplant site was 185.8±33.8 and 195.2±40.6 g DW m^−2^, respectively (Figure 4). At the 2010 transplant site, the maximum aboveground biomass of reproductive and vegetative shoots was 197.4±35.7 and 263.2±54.7 g DW m^−2^, respectively. Statistical analysis showed that there were no significant differences between the 2009- and the 2010-transplant sites in shoot density, shoot height and aboveground biomass (all p>0.05). C, N and P contents of eelgrass leaf collected on June 19, 2012 at the 2009 site were 36.42±1.86%, 2.26±0.13%, and 0.31±0.05%, respectively.

Shoot height and biomass of both reproductive and vegetative shoots of the transplanted eelgrass first increased and then decreased. Shoot height and biomass reached their highest level at the end of spring. The vegetative shoots in summer maintained relatively high levels of height and aboveground biomass, but in autumn both parameters began to decrease rapidly. The variations in height and aboveground biomass of the transplanted eelgrass were basically consistent with that of water temperature.

At the plots used to test the simplified transplant method in which each stone-anchored PU was placed on the surface of the tidal flat, one month survivorship averaged 85.7±7.4% and two month survivorship was 83.3±9.8%. These values were significantly lower (p<0.01) than those at the 2009 and 2010 sites, in which the regular stone anchoring method was used. However, after this initial loss of plants in the simplified transplant method plot, the transplants became established and shoot density increased via lateral shoot production from the transplants. On June 20, 2012, the shoot density reached 348±51 shoots m^−2^ and shoot height averaged 71.2±26.8 cm; both of these values were similar to those at the 2009 and 2010 transplant sites (one-way ANOVA; p>0.05).

Table 2 shows the shoot density, shoot height and aboveground biomass of the natural eelgrass bed (donor site for 2010 transplantation) in the intertidal zone of Qingdao Bay. On 19 June 2012, the average shoot height of the vegetative and reproductive shoots was 86.0±16.9 and 103.8±22.3 cm, respectively; and the aboveground biomass of the vegetative and reproductive shoots was 235.2±40.1 and 131.3±27.5 g DW m^−2^. All data determined on 19 June 2012 (after adult-plant collection) were statistically the same as those obtained on 21 June 2009 (before adult-plant collection; all p>0.05), indicating our adult-plant-collection practice had little impact on the donor meadow. Statistical analysis showed that there were no significant differences between the transplant sites (2–3 years after transplantation) and the reference meadow (Qingdao bay) in shoot density, shoot height and aboveground biomass (all p>0.05).

**Table 2:** Shoot density, shoot height, and aboveground biomass (AG-biomass) for a natural intertidal *Zostera marina* bed (Qingdao Bay) monitored on June 21, 2009 (A) and June19, 2012 (B). Values are means (±SD).

		Vegetative shoot			Reproductive shoot		
		Shoot density (shoots m^−2^)	Shoot height (cm)	AG-biomass (g DWm^−2^)	Shoot density (shoots m^−2^)	Shoot height (cm)	AG-biomass (g DW m^−2^)
A	Mean	311	86.0	235.2	106	103.8	131.3
	SD	(88)	(16.9)	(40.1)	(21)	(22.3)	(27.5)
B	Mean	302	87.7	165.6	102	104.1	155.4
	SD	(91)	(13.8)	(45.4)	(26)	(20.7)	(41.9)

**Figure 5:**
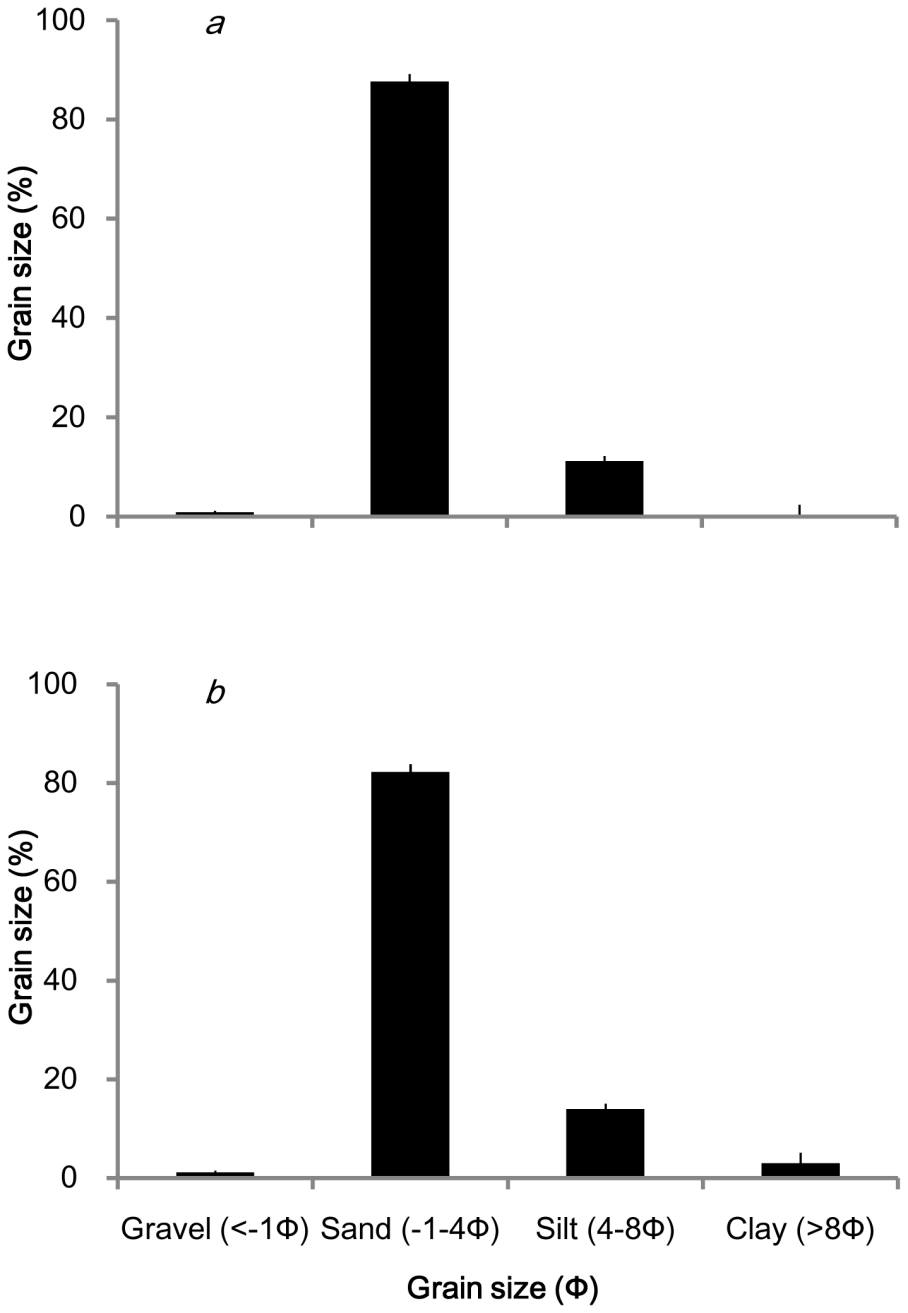
Particle size spectra of sediments in natural (*a*; Huiquan Bay) and transplanted *Zostera marina* areas (*b*; Qingdao Bay). Values are means ± SD.

As shown in Figure 5, the average particle size of the sediments in the natural eelgrass bed in Qingdao Bay was 3.25Ф. The sediment was dominated by sand (mean 82.27%). The average particle size of sediments at the transplant site in Huiquan Bay was 2.82Ф, and the sediment was also dominated by sand (mean 87.64%). Comparatively, there were more fine particles (silt and clay; 17.04%) and less sand in the Huiquan Bay samples than in the Qingdao Bay samples (p<0.05).

## Discussion

This study showed that there were no significant differences between the transplant sites (2–3 years after transplantation) and the reference meadow (Qingdao bay) in shoot density, shoot height and aboveground biomass (all p>0.05). At all sites, values of these parameters first increased and then decreased. They reached their maximum values from June to July, indicating good growth conditions. The seasonal variations of shoot height and aboveground biomass are summarized as follows: In spring when the water temperature was optimal, the eelgrass plants grew rapidly. In early summer, the number of eelgrass plants reached the maximum level. When the water temperature exceeded the optimal level (i.e. 15–20 °C) and when the plants were exposed to sunlight during ebb tide, a massive number of epiphytic algae appeared [36]. Under these conditions, the growth of eelgrass was inhibited, and aging and disappearance of old shoots were accelerated. Shoot height and biomass began to decline, and minimum levels were found winter, when the water temperature was low. However, vegetative reproduction was high in autumn and aboveground biomass was maintained at a certain level.

Observations of the experimental plots revealed that reproductive shoots of the transplanted eelgrass began to grow in April, and this marked the beginning of sexual reproduction. The reproductive shoots disappeared in August, which marked the end of sexual reproduction. Thus, the vegetative reproduction period of the transplanted eelgrass in Huiquan Bay was from April to August. Reproductive shoots appeared in the natural eelgrass bed in Qingdao Bay in mid–late March, 2009, and by late July they had mostly disappeared. Therefore, the sexual reproduction period of the natural eelgrass bed in Qingdao Bay was from March to July. These data indicate that the sexual reproduction period of eelgrass in Huiquan Bay in 2012 was delayed, and this delay likely was due to cooler water temperatures. The water temperature of the intertidal zone in Qingdao Bay was 18, 20.1, and 22.3 °C on April 4, May 23, and June 21, 2009, respectively. In contrast, on April 19, May 19, and June 19 of 2012, the water temperature was 9.4, 13.3, and 17.7 °C, respectively, in the intertidal zone of Huiquan Bay.

The shoot density of the transplanted eelgrass significantly increased at two time points during the observation period. The first increase likely resulted from vegetative reproduction under the optimal environmental conditions during spring. In mid-September, lateral shoots appeared in massive numbers, indicating that vegetative reproduction was dominant. In late October, seedlings that had developed from seed germination were found in the transplanted bed, but the density was low and plants were difficult to count. For most seagrass species, vegetative reproduction plays an important role in maintaining the seagrass bed [37]. It has been shown that the loss rate of seeds and seedlings produced by sexual reproduction in natural seagrass beds can be as high as 90% [38]–[39]. Hence, the expansion of the transplanted eelgrass in the present study area was due mainly to vegetative reproduction. Generally, vegetative reproduction occurs throughout the year. However, due to environmental factors, a certain seasonal pattern was identified in this study, with vegetative reproduction being more prevalent in autumn.

Sediment particle size is an important factor that influences the growth of seagrass rhizomes [40]–[41]. Rhizome internodes grow rapidly in sediments with small particle size. Bos et al. [42] showed that eelgrass plants could improve their growth environment by promoting sediment deposition. The natural eelgrass plants in Qingdao Bay are mainly distributed in middle and lower areas of the intertidal zone. Thus, the transplantation experiment was conducted in the intertidal zone of Huiquan Bay.

Various anchoring devices, including staples, nails, rods, shells, wire mesh, and TERFS (transplanting eelgrass remotely with frame systems), have been designed in attempts to develop effective transplanting techniques [16], [22], [43]–[44]. In our stone anchoring method, why the buried PU rhizomes were situated on the side of, but not underneath, the anchoring stone is to minimize the possibility of sulfide accumulation that is toxic and may result in seagrass mortality [45]–[47]. The stone anchoring method used in the eelgrass transplantation trials in the present study was successful, and this technique offers great advantages for use in restoration efforts. Firstly, the success rate of eelgrass transplantation was extremely high. The three month survivorship was nearly 100%. After 2 to 3 years, the shoot height and biomass of the transplanted plants was very similar to those of the nearby natural population. Secondly, the stone anchoring method is convenient and environmentally friendly. Given that the gravel is collected from the marine environment and the cotton thread is biodegradable, this method leaves nothing hazardous in the transplanting area after restoration. Thirdly, this method is fast. The estimated total time to plant one PU using this method, including plant collection, sorting, stone binding, and transplanting, is about 60 seconds per PU; transplanting of each PU takes about 5 seconds. In addition, our modified stone anchoring method involved simply placing each stone-anchored PU on the surface of the tidal flat is a more simple and feasible technique with considerable success. This modified method, we believe, would be convenient for transplantation at shallow subtidal sites, for example, in the subtidal zone with water depth less than 0.5 m at low tide. Also, the simplified method would be easy for SCUBA diver to implement seagrass transplanting in the subtidal zone. It is suggested that the method might be more effective for muddy seabed.

Davis & Short [16] developed the horizontal rhizome method in which two shoots in opposite directions are secured horizontally into the sediment using a bamboo staple; and transplantation in the intertidal and subtidal zones results in high survival rates of 75–99% after a year. The transplantation process takes only takes 5.8 seconds per PU. However, this method requires more time and manpower, especially for shoot transplantation in the subtidal zone. Orth et al. [17] used a method in which a single unanchored shoot is inserted directly into the sediments at a depth of 2.5–5.0 cm. Implementation of this method (shoot collection, sorting, and transplanting) takes as little as 21 seconds per PU, but one month survival of the planted eelgrass is only 73%. At the initial transplant trial, we also considered of this shoot unanchored method to transplant eelgrass in the intertidal zone of Huiquan Bay, but we found strong winds and waves might uproot the unanchored shoots and result in great loss of the transplanted plants. Also, Orth et al. [17] pointed out that use of anchors may be more appropriate with sites that receive frequently storms. In summary, the stone anchoring method described herein is a promising new technique for use in eelgrass restoration projects.

## Supporting Information

File S1 Supporting information.
(DOCX)

## References

[1] Short FT, Wyllie-Echeverria S (1996) Natural and human-induced disturbance of seagrasses. Environmental conservation 23: 17–27.

[2] Costanza R, d'Arge R, de Groot R, Farber S, Grasso M, et al. (1997) The value of the world's ecosystem services and natural capital. Nature 387: , 253–260.

[3] Duarte CM, Borum J, Short FT, Walker DI (2008) Seagrass Ecosystems: Their Status and Prospects. In: N.V.C. Polunin, (Ed.) Aquatic Ecosystems: Trends and Global Prospects. Cambridge University Press. pp. 281–294.

[4] Bouma TJ, De Vries MB, Low E, Peralta G, Tánczos IC, et al (2005) Trade-offs related to ecosystem engineering: A case study on stiffness of emerging macrophytes. Ecology 86: 2187–2199.

[5] van der Heide T, Eklöf JS, van Nes EH, van der Zee EM, Donadi S, et al (2012) Ecosystem Engineering by Seagrasses Interacts with Grazing to Shape an Intertidal Landscape. PLoS One 7(8): e42060 10.1371/journal.pone.0042060 22905115 PMC3414520

[6] Heck KL Jr, Valentine dJF (2006) Plant-herbivore interactions in seagrass meadows. Journal of Experimental Marine Biology and Ecology 330: 420–436. 10.1016/s0022-0981(00)00342-7 11239626

[7] Barbier EB, Hacker SD, Kennedy C, Koch EW, Stier AC, et al (2011) The value of estuarine and coastal ecosystem services. Ecological Monographs 81: 169–193.

[8] Liu XJ, Zhou Y, Yang HS, Ru SG (2013) Eelgrass detritus as a food source for the sea cucumber *Apostichopus japonicus* Selenka (Echinidermata: Holothuroidea) in coastal waters of north China:an experimental study in flow-through systems. PLoS ONE 8(3): e58293 10.1371/journal.pone.0058293 23505480 PMC3591415

[9] Fourqurean JW, Duarte CM, Kennedy H, Marba N, Holmer M, et al (2012) Seagrass ecosystems as a globally significant carbon stock. Nature Geoscience 5: 505–509.

[10] Greiner JT, McGlathery KJ, Gunnell J, McKee BA (2013) Seagrass Restoration Enhances “Blue Carbon” Sequestration in Coastal Waters. PLoS ONE 8(8): e72469 10.1371/journal.pone.0072469 23967303 PMC3743776

[11] Lavery PS, Mateo M-Á, Serrano O, Rozaimi M (2013) Variability in the Carbon Storage of Seagrass Habitats and Its Implications for Global Estimates of Blue Carbon Ecosystem Service. PLoS ONE 8(9): e73748 10.1371/journal.pone.0073748 24040052 PMC3764034

[12] Orth RJ, Carruthers TJB, Dennison WC, Duarte CM, Fourqurean JW, et al. (2006) A global crisis for seagrass ecosystems. Bioscience 56: , 987–96.

[13] Waycott M, Duarte CM, Carruthers TJB, Orth RJ, Dennison WC, et al (2009) Accelerating loss of seagrasses across the globe threatens coastal ecosystems. Proceedings of the National Academy of Sciences of the United States of America 106: 12377–12381. 19587236 10.1073/pnas.0905620106 PMC2707273

[14] Short FT, Polidoro B, Livingstone SR, Carpenter KE, Bandeira S, et al (2011) Extinction risk assessment of the world's seagrass species. Biological Conservation 144: 1961–1971.

[15] Phillips RC (1974) Transplantation of seagrasses, with special emphasis on eelgrass, *Zostera marina* L. Aquaculture. 4: 161–176.

[16] Davis RC, Short FT (1997) Restoring eelgrass, *Zostera marina* L., habitat using a new transplanting technique: The horizontal rhizome method. Aquatic Botany 59: 1–15.

[17] Orth RJ, Harwell MC, Fishman JR (1999) A rapid and simple method for transplanting eelgrass using single, unanchored shoots. Aquatic Botany 64: 77–85.

[18] van Katwijk MM (2000) Reintroduction of eelgrass (*Zostera marina* L.) in the Dutch Wadden Sea: a research overview and management vision. In Wolff WJ, Essink K, Kellermann A, van Leeuwe MA, editors. Challenges to the Wadden Sea. Proceedings of the10th International Scientific Wadden Sea Symposium, Groningen. Ministry of Agriculture, Nature Management and Fisheries. University of Groningen, Dept. of Marine Biology, 2003.

[19] Paling EI, van Keulen M, Wheeler K, Phillips J, Dyhrberg R (2001) Mechanical seagrass transplantation in Western Australia. Ecological Engineering 16: 331–339.

[20] Fishman JR, Orth RJ, Marion S, Bieri J (2004) A comparative test of mechanized and manual transplanting of eelgrass, *Zostera marina*, in Chesapeake Bay. Restoration Ecology 12: 214–219.

[21] Bell SS, Tewfik A, Hall MO, Fonseca MS (2008) Evaluation of seagrass planting and monitoring techniques: implications for assessing restoration success and habitat equivalency. Restoration Ecology 16: 407–416.

[22] Lee KS, Park JI (2008) An effective transplanting technique using shells for restoration of *Zostera marina* habitats. Marine Pollution Bulletin 56: 1015–1021. 18342894 10.1016/j.marpolbul.2008.02.010

[23] Busch KE, Karrh LP, Golden RR, Lewandowski MJ, Parham TA, et al (2010) Large-scale *Zostera marina* (eelgrass) restoration in Chesapeake Bay, Maryland, USA. Part I: a comparison of techniques and associated costs. Restoration Ecology 18: 490–500.

[24] Golden RR, Busch KE, Karrh LP, Parham TA, Lewandowski MJ, et al. (2010) Large-Scale *Zostera marina* (eelgrass) Restoration in Chesapeake Bay, Maryland, USA. Part II: A Comparison of Restoration Methods in the Patuxent and Potomac Rivers. Restoration Ecology 18: 501–513.

[25] Shafer DJ, Bergstrom P (2010) An introduction to a special issue on large-scale submerged aquatic vegetation restoration research in the Chesapeake Bay: 2003–2008. Restoration Ecology 18: 481–489.

[26] Balestri E, Lardicci C (2012) Nursery-propagated plants from seed: a novel tool to improve the effectiveness and sustainability of seagrass restoration. Journal of Applied Ecology 49: 1426–1435.

[27] Cunha AH, MarbàNN, van Katwijk MM, Pickerell C, Henriques M, et al (2012) Changing paradigms in seagrass restoration. Restoration Ecology 20: 427–430.

[28] Harwell MC, Orth RJ (1999) Eelgrass (*Zostera marina* L.) seed protection for field experiments and implications for large-scale restoration. Aquatic Botany 64: 51–61.

[29] Orth RJ, Marion SR, Granger S, Traber M (2009) Evaluation of a mechanical seed planter for transplanting *Zostera marina* (eelgrass) seeds. Aquatic Botany 90: 204–208.

[30] Fonseca MS, Kenworthy WJ, Courtney FX, Hall MO (1994) Seagrass planting in the Southeastern United States: methods for accelerating habitat development. Restoration Ecology 2: 198–212.

[31] Bastyan GR, Cambridge ML (2008) Transplantation as a method for restoring the seagrass *Posidonia australis* . Estuarine, Coastal and Shelf Science 79: 289–299.

[32] Park JI, Lee KS (2010) Development of transplantation method for the restoration of surfgrass, *Phyllospadix japonicus*, in an exposed rocky shore using an artificial underwater structure. Ecological Engineering 36: 450–456.

[33] Short F, Carruthers T, Dennison W, Waycott M (2007) Global seagrass distribution and diversity: A bioregional model. Journal of Experimental Marine Biology and Ecology 350: 3–20.

[34] Solórzano L, Sharp JH (1980) Determination of total dissolved phosphorus and particulate phosphorus determination in natural waters. Limnol & Oceanogr 25: 754–758.

[35] Zhou Y, Zhang FS, Yang HS, Zhang SM, Ma XN (2003) Comparison of effectiveness of different ashing auxiliaries for determination of phosphorus in natural waters, aquatic organisms and sediments by ignition method. Water Res 37: 3875–3882. 12909105 10.1016/s0043-1354(03)00267-7

[36] Lee KS, Park SR, Kim YK (2007) Effects of irradiance, temperature, and nutrients on growth dynamics of seagrasses: A review. Journal of Experimental Marine Biology and Ecology 350: 144–175.

[37] Hemminga MA, Duarte CM (2000) Seagrass ecology: Cambridge University Press.

[38] Fishman JR, Orth RJ (1996) Effects of predation on *Zostera marina* L. seed abundance. Journal of Experimental Marine Biology and Ecology 198: 11–26.

[39] Orth RJ, Harwell MC, Bailey EM, Bartholomew A, Jawad JT, et al (2000) A review of issues in seagrass seed dormancy and germination: implications for conservation and restoration. Marine Ecology Progress Series 200: 277–288.

[40] Short F, Davis RC, Kopp BS, Short CA, Burdick DM (2002) Site-selection model for optimal transplantation of eelgrass *Zostera marina* in the northeastern US. Marine Ecology Progress Series 227: 253–267.

[41] Bradley MP, Stolt MH (2006) Landscape-level seagrass–sediment relations in a coastal lagoon. Aquatic botany 84: 121–128.

[42] Bos AR, Bouma T, de Kort GLJ, van Katwijk MM (2007) Ecosystem engineering by annual intertidal seagrass beds: Sediment accretion and modification. Estuarine, Coastal and Shelf Science 74: 344–348.

[43] Phillips RC, McRoy CP (1990) Seagrass research methods. Unesco.

[44] West RJ, Jacobs NE, Roberts DE (1990) Experimental transplanting of seagrasses in Botany Bay, Australia. Marine Pollution Bulletin 21: 197–203.

[45] Larkum AWD, Orth RJ, Duarte CM (2006) Seagrasses: Biology, Ecology, and Conservation. Springer, Berlin.

[46] Calleja ML, Marba N, Duarte CM (2007) The relationship between seagrass (*Posidonia oceanica*) decline and sulfide porewater concentration in carbonate sediments. Estuarine, Coastal and Shelf Science 73: 583–588.

[47] Koch MS, Schopmeyer S, Kyhn-Hansen C, Madden CJ (2007) Synergistic effects of high temperature and sulfide on tropical seagrass. Journal of Experimental Marine Biology and Ecology 341: 91–101.

