# Supplementary material for: Restoring Eelgrass (Zostera marina L.) Habitats Using a Simple and Effective Transplanting Technique
Source: PLoS One. 2014 Apr 2;9(4):e92982. doi: 10.1371/journal.pone.0092982 (PMC3973628; doi:10.1371/journal.pone.0092982)
Supplement: File S1 — Supporting information. (DOCX) [file pone.0092982.s001.docx]

A


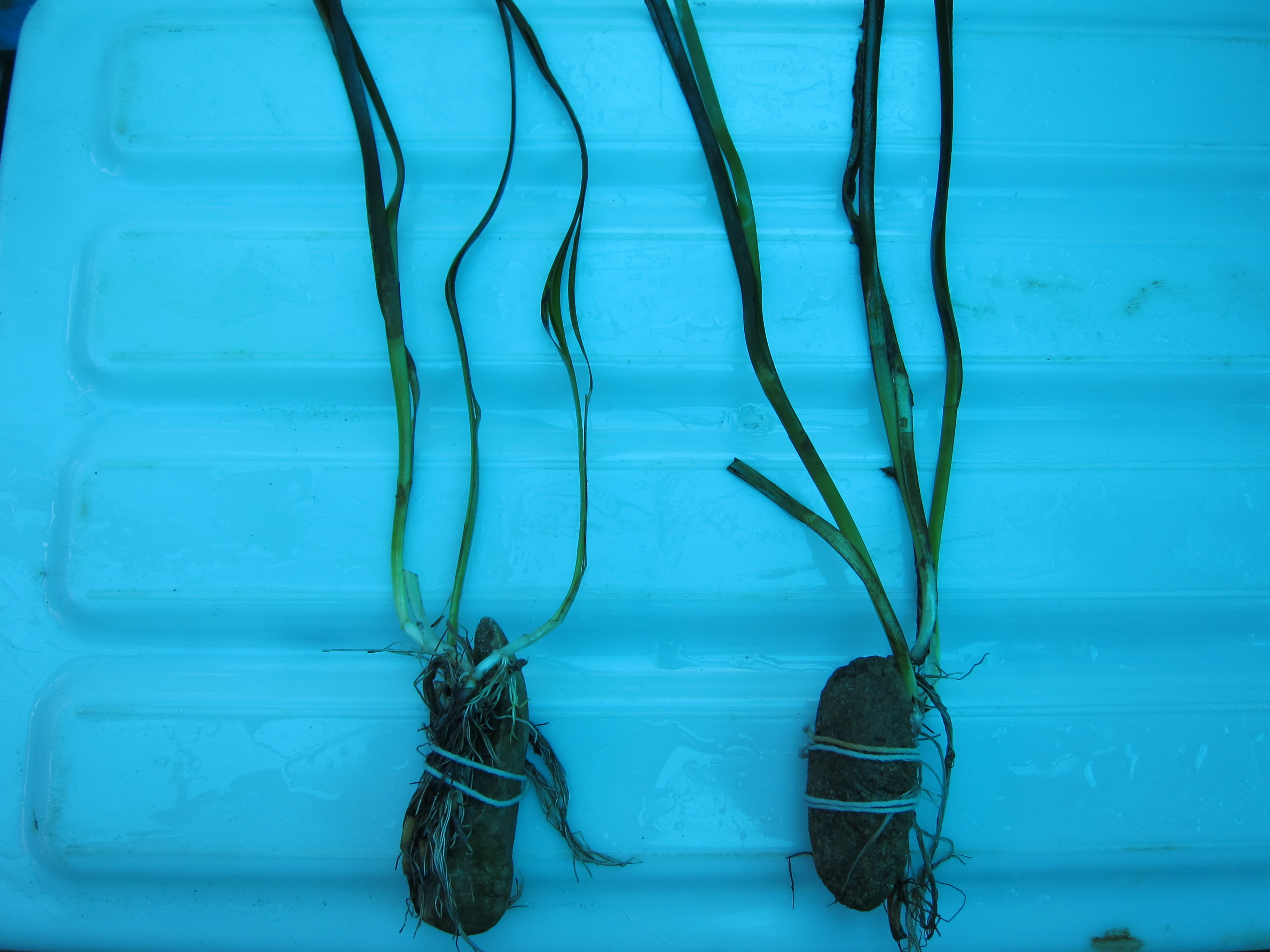


B


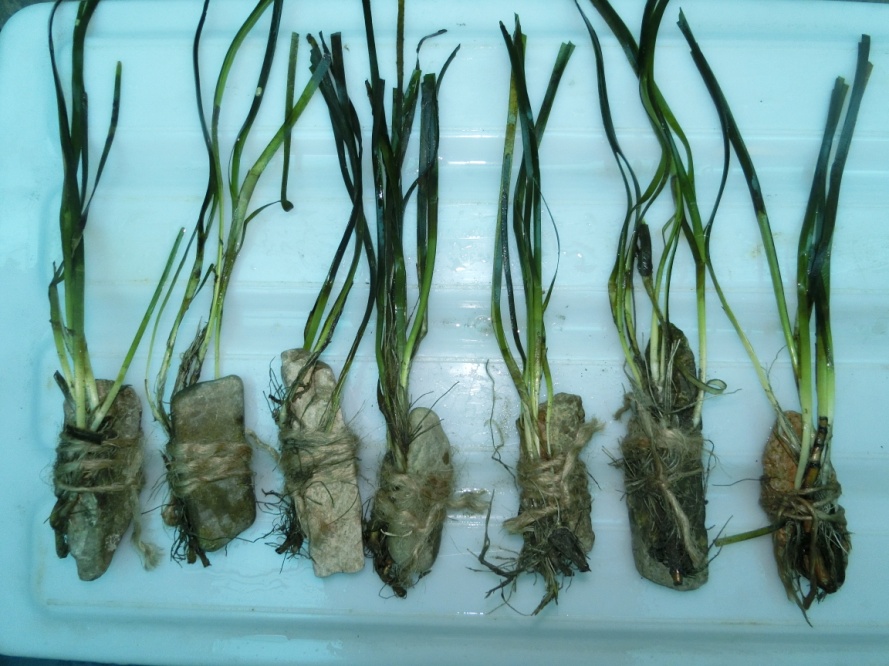


Fig.1. A photo showing stones with shoots; for each small elongate stone of 50–150 g, three shoots with rhizomes and roots were tied using biodegradable cotton (A) or hemp (B) thread.


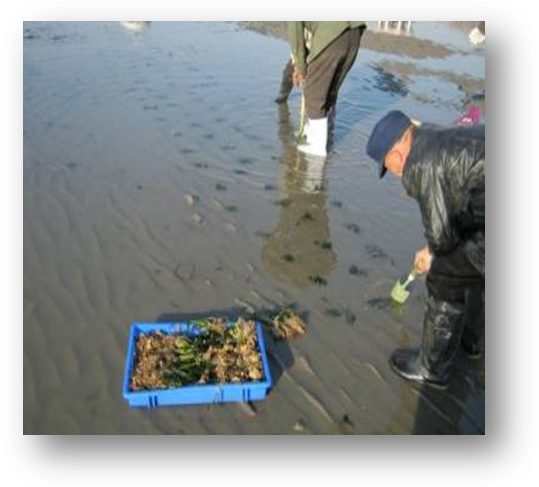


Fig.2. A photo showing eelgrass transplanting process. PUs were buried in holes dug with a scoop so that the rhizomes were situated at a depth of 2–4 cm in sediments.
